# Moving beyond Inclusion to Belonging

**DOI:** 10.3390/ijerph20206907

**Published:** 2023-10-10

**Authors:** Toby Long, Jennifer Guo

**Affiliations:** Center for Child and Human Development, Georgetown University, 2115 Wisconsin Avenue NW, Washington, DC 20007, USA

**Keywords:** children with disabilities, inclusion, participation, belonging

## Abstract

This paper explores the concepts of inclusion, participation, and belonging in the context of development for children with disabilities. The importance of creating an environment that embraces diversity, encourages active engagement, and nurtures a sense of belonging for children is discussed. The authors provide insights into the benefits of inclusive practices, strategies to enhance participation, and methods to foster a sense of belonging in children with disabilities. The authors argue that service providers and service systems must move beyond fostering social inclusion and inclusive education, although emphasized globally, and focus on promoting participation and ultimately belonging to ensure that children with disabilities are full members of their communities.

## 1. Introduction

It is estimated that over one billion people, about 16% of the world’s population, have a disability [1]. Article 1 of the United Nations Convention on the Rights of Persons with Disabilities (CRPD) [2] defines individuals with disabilities as ‘those who have long-term physical, mental, intellectual or sensory impairments which in interaction with various barriers may hinder their full and effective participation in society on an equal basis with others.’ Barriers, as mentioned in the CRPD, encompass physical barriers, such as inaccessible infrastructure; social barriers, such as prejudice against those with disabilities and stigma; and legal or regulatory barriers, such as the inability of disabled adults to adopt children. Whether these barriers are physical aspects of the landscape, exclusionary policies, or attitudinal, individuals with disabilities have historically been segregated from the general population as a result.

Out of the 1.3 billion individuals who experience disability, approximately 240 million are children [3]. Using data from 195 countries and territories, the 2018 Global Burden of Diseases, Injuries, and Risk Factors Study (GBD) [4] estimated that 53 million children younger than 5 years live with developmental disabilities worldwide, with approximately 95% living in low and middle-income countries (LMIC). Additional data from the 2019 GBD study [5] indicated that the probability of a child experiencing a disability before reaching the age of five was 10 times higher than the likelihood of mortality.

The 2022 UNICEF Fact Sheet for Children with Disabilities [6] reports that children with disabilities face higher levels of discrimination and encounter significant challenges in their educational development. In particular, compared to their peers without disabilities, students with disabilities often exhibit substantially poorer foundational reading and numeracy skills and are more likely to be out of school or never start. They are also less likely to complete primary education even when given the opportunity to attend school due to the wide learning gaps between them and their non-disabled peers [7]. The educational disparities between children with disabilities and those without are accentuated by poverty as individuals with disabilities, including children, are overrepresented in the poorest segments of society.

According to the World Bank [8], approximately 85% of primary school-age children with disabilities who are not enrolled in school have never attended school. Data [7] also suggest that the exclusion of individuals with disabilities from meaningful participation in society, including educational and employment opportunities, may result in a reduction in a nation’s GDP by 3 to 7 percent. Investing in early care and education for all children, including those with disabilities, can lead to substantial returns. Garcia and colleagues [9] showed that there is a 13% return on investment (ROI) for quality early childhood programs. Excluding children from education not only harms the individual child by diminishing their earning potential and increasing their social service needs but also has far-reaching consequences for society. Inclusive and quality education for all children, ensuring their unhindered participation in society, is a matter of individual rights and a collective responsibility that benefits everyone. Including children in education and all other aspects of community life is crucial for fostering social cohesion, promoting equal opportunity, and contributing to society’s overall well-being and prosperity. To address the problem of how best to ensure that all children, regardless of their ability, have the opportunity to participate in society requires the field to examine the concept of inclusion and to question if inclusion is the ultimate goal. The intent of this paper is to explore the concept of inclusion and to propose that this field moves toward ensuring that children have the opportunities to participate in society and create a sense of belonging.

## 2. The International Community’s Commitment to Including People with Disabilities

Individual countries and the global community have made commitments to the inclusion of people with disabilities, including children, in all aspects of the community. The United States has been at the forefront of this movement as it has established three laws to protect and support individuals with disabilities, promote their rights, and ensure equal opportunities (Table 1).

Recent years have also seen increased international efforts to support the inclusion of people with disabilities and, specifically, children with disabilities. In 2001, the International Classification of Function, Disability, and Health (ICF) [13] was officially endorsed by all 191 Member States of the World Health Organization (WHO) as the international standard for describing and measuring health and disability. The ICF [13] provides a comprehensive understanding of health and disability, acknowledging that the interaction between an individual’s health condition and the environment can enable or hinder their participation and functioning.

The United Nations Disability Inclusion Strategy [14] is a policy and accountability framework that is used to promote change and assess progress on inclusion regarding disabilities. The Strategy [14] requires that disability and intersectionality be considered in all aspects of UN programming, and it fosters collaboration among groups to accelerate progress and achieve inclusion. The Strategy [14] supports the UN’s implementation of other international inclusive efforts, such as the CRPD and the Convention on the Rights of the Child (Table 2).

In addition to the United Nations, the World Bank 2019 launched the Inclusive Education Initiative (IEI) [8], a multi-donor trust fund that can be used to support countries providing inclusive education for children with disabilities. IEI [8] acknowledges the importance of inclusive education to achieve the broader goals of quality education, social inclusion, and poverty reduction. Despite this clear global commitment to individuals with disabilities, success in ensuring an inclusive and equitable international society remains elusive.

## 3. Mainstreaming to Belonging: What Do We Mean?

As discussed above, the international community has embraced the concept of inclusion and has created a variety of treaties, conventions, regulations, statements, programs, etc., to promote inclusion. Given that many children with disabilities continue to be excluded from community activities or are never afforded the services, accommodations, or modifications necessary to be included, it is unclear if there is a universal understanding of inclusion and if inclusion should be considered the ultimate goal. The next section provides operational definitions of various concepts that intersect and expand upon inclusion.

*Mainstreaming* describes placing children with disabilities in regular or “mainstream” settings alongside their non-disabled peers. It emphasizes the physical integration of children with disabilities into traditional classrooms or other spaces rather than segregating them into separate “special” settings or programs. Children who are “mainstreamed” often receive specialized services or support outside of the mainstream. For example, a child who is mainstreamed into a classroom for typically developing children may receive specialized instruction in a resource room and specialized therapeutic services outside the classroom, thus spending most of their day outside the mainstream classroom.

*Inclusion* reflects a shift in focus from physical integration to a broader approach that promotes equal access and support for all children, regardless of their abilities. Inclusion emphasizes the creation of inclusive environments that address the diverse needs of all children. Inclusive education, for example, requires children with disabilities to be educated in the same classroom as their non-disabled peers. Inclusion, as described by Karlsudd [22], requires special efforts; services and support are needed to promote the success of every person, regardless of their abilities. The specialized services and supports needed are offered as part of the regular classroom. Support is also provided to the classroom teacher to ensure all lessons are differentiated, curricula are adapted or modified, and accommodations are provided to meet the unique needs of each student within the classroom environment.

Nilholm as described by Maxwell, et al. (2018) [23] proposed three perspectives on inclusion. The Compensation perspective, which is the most common and similar to the Medical Model of Disability, emphasizes a child’s impairment or disability as the reason for exclusion. The focus is on identifying the child’s limitations and providing individualized accommodations or remediation services to enable them to be included. The Critical Perspective [23] is comparable to the Social Model of Disability as it identifies this problem within a broader context, such as the school environment. The focus is on changing structures, policies, and attitudes in the system to remove barriers and promote equal access for all. Nilholm’s third perspective, the Dilemma Perspective [23], is a critique of the Compensation and Critical perspectives, recognizing participation as a critical element that needs to be addressed to ensure meaningful inclusion. This perspective acknowledges that systems face inherent dilemmas when striving for inclusion. A fundamental dilemma for educational systems is categorizing children as either deficient or unique individuals. Finding a balance between categorization and individualization is critical to remediate, support, or accommodate as appropriate; such a balance is also necessary to value and accept each child’s unique characteristics. This may involve adopting inclusive practices that recognize and celebrate the diversity of all children while also providing tailored interventions and accommodations as needed so that students with disabilities can participate inside and outside of the classroom.

**Figure 1:**
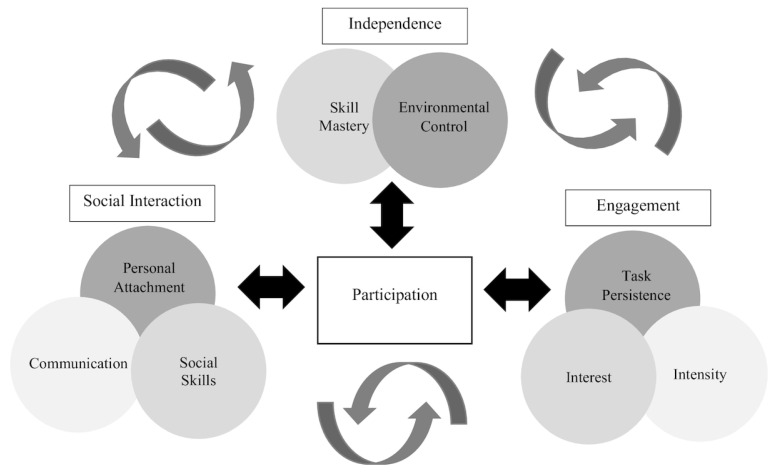
The multidimensionality of participation and the interactions between its components highlight the inherent complexity of defining and understanding participation for young children [25].

According to the ICF [13], *participation* is defined as involvement in life situations with family, same-aged peers, and other community members and is distinct from inclusion. The ICF [13] conceptualizes disability by considering the interplay between multiple factors, including personal and environmental factors, body structure and function, and engagement in activities and participation. Consequently, participation is a multidimensional construct that encompasses various aspects of independent performance and engagement in activities and routines that are preferred by and meaningful for the individual (Figure 1). Participation requires availability and access to environments and activities at the same frequency seen in non-disabled peers and engagement in those activities [24]. Participation occurs in the presence of and with others, highlighting the importance of social interactions and connections in this process. To promote a child’s participation, supportive services can be targeted at any of the following three components [25]: independence, social interaction, and engagement (Figure 1).

*Belonging* is when a person feels an emotional connection to a community. The conceptual description offered by Mahar, Cobigo, and Stuart [26] pertains to children with disabilities. Following an extensive scoping review, they identified five intersecting themes that research related to belonging has refined specifically to people with disabilities. The themes of subjectivity, groundedness to an external referent (school, peer group, community, etc.); reciprocity; self-determination; and dynamism indicate that belonging is a dynamic phenomenon that can be hindered or promoted by the environment and interpersonal relationships. Contemporary research, building upon this framework, indicates that belonging is fostered by promoting inclusion and participation but is also distinct from this and requires both to be realized, as is discussed next.

Similar to Mahar et al. Allen and colleagues [27] define belonging as the subjective perception or feeling one experiences when surrounded by family, friends, school, work environments, communities, cultural groups, and physical places. Belonging can be promoted or hindered by people, places, and experiences that interact with an individual’s characteristics, culture, past experiences, and identity [27]. Thus, belonging can occur in a variety of contexts as long as that context provides the appropriate support. Allen [27] suggests that belonging is made up of four interrelated components that are supported by systems with which the person interacts: competencies for belonging (skills and abilities); opportunities to belong (enablers, removal/reduction in barriers); motivations to belong (inner drive); and perceptions of belonging (based on past experiences). Systems can support or hinder the expression of these components.

Based on the interviews of 24 youths (aged 13–24 years) with various intellectual and developmental disabilities, Renwick and colleagues [28] found that the participants identified belonging as closely linked to friendship, the experience of being included in their communities, and was something they actively sought out and considered crucial in their lives. The researchers [28] identified four components of belonging: shared interests, navigating social norms, negotiating meaningful roles, and engaging in social relationships. Most participants [28] emphasized that the presence or absence of social relationships played a significant role in shaping their sense of belonging. Having meaningful connections with family, friends, and community members contributed positively to their overall sense of belonging; conversely, a lack of quality relationships bred feelings of isolation. Having the same disability is insufficient to sustain meaningful friendships; engaging with others who share similar interests, regardless of disability, significantly strengthens feelings of belonging. Many participants [28] emphasized the importance of being part of the community via meaningful contributions such as attending, working, or volunteering at schools, workplaces, religious organizations, neighborhood online groups, etc., as a crucial aspect of belonging. Finally, the participants [28] described the complex process of negotiating their own desires and aspirations while navigating social norms and the expectations of others, including family, teachers, and service providers.

Seligman [29] proposed that belonging can be distinguished from participation because an individual feels like they belong when they derive meaning and purpose from being a part of and serving something bigger than oneself. The sense of connection and feeling valued and appreciated are critical components of belonging.

## 4. The Role of Service Providers in Fostering Belonging

As demonstrated by international and individual country commitments, inclusion appears to be the ultimate outcome of services and support for children with disabilities. Although inclusion is critical in order to create a more equitable society, we must move beyond inclusion to facilitate a child’s participation in the community and ultimately foster a sense of belonging among individuals with disabilities. In this context, belonging, as discussed above, requires the interaction between skill and the mastery of activity, advocacy, and social connectedness. This broader understanding of belonging encompasses multiple dimensions that are essential for promoting the well-being of individuals with disabilities and is consistent with the components proposed by Mahar et al. [26].

Shifting the focus from inclusion to participation and ultimately to a sense of belonging for children with disabilities necessitates a shift in the approach taken by providers when serving children and their families. Fostering belonging requires service providers to consider and understand the wants and desires of the children and their families regarding the services, support, and spaces with which they engage. This change opens up the possibility of diversifying approaches to service provision to better meet the individual needs and preferences of children with disabilities and shifts the focus from impairments, as promoted by the ICF [13].

**Figure 2:**
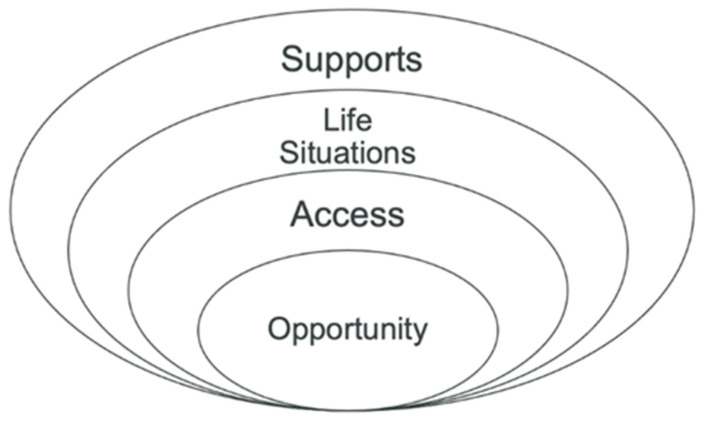
Necessary components of participation and belonging.

This shift in service provision includes the maximization of learning, social, and activity opportunities for children with disabilities and the changing role of service providers. Three factors impact this shift in service provision for children with disabilities, as well as the professional development of service providers. (Figure 2). Providing children who have disabilities with *opportunities* to be with non-disabled peers and engage in meaningful and age-appropriate activities is essential for their development, growth, inclusion, participation, and belonging. Children should not be rejected or told they are “not ready” based on preconceived notions or assumptions about their abilities, their diagnosis, or their impairments. Opportunities for activity, socialization, and learning that are available to non-disabled peers should be available and welcoming to children with disabilities. Once opportunities are provided, it is crucial to ensure that children with disabilities can *access* the *life situations* they desire or are expected to be involved in, which are afforded by these opportunities. 

Opportunities in various activities, routines, and environments provide sufficient intensity and frequency to foster the repetition and practice needed for growth and development to occur and allow children and families to make choices based on their priorities and interests [30]. The United States federal regulations, such as IDEA [11] and Section 504 [10]. mandate that all children, regardless of their disability, are educated and serve in the least restrictive environment. Internationally, the UN Convention on the Rights of the Child [15], which most countries have signed on to, promotes community-based inclusion for all children. No child should be denied opportunities based on their disability. The role of this service provider is to ensure that children with disabilities have *access* to the appropriate support, accommodations, adaptations, and modifications that enable their growth and development within these opportunities.

Access extends beyond physical access. Children with disabilities should be able to access the information, activities, and programming that occurs within a space like a classroom, ball field, scout meeting, etc. Implementing Universal Design and Universal Design for Learning (UDL) principles can significantly enhance the experience of children with disabilities and support access, inclusion, and participation by providing multiple means of representation, expression, and engagement to meet the diverse needs of all children. Also, assistive technology can enable children with disabilities to participate more fully in all aspects of life, helping them access information and activities more effectively and efficiently [31].

Engaging in *life situations* requires accessible environments, programs, and activities and is promoted by the International Classification of Functioning [13]: a framework that service providers worldwide have adopted. It is essential for service providers to support children and families in identifying the most meaningful life situations, implementing individualized interventions to promote a child’s participation in those situations, and developing environmental support to ensure a child’s participation. Life situations include participating in entertainment, hobbies, family life, religious activities, etc., in addition to activities of daily living, such as dressing, eating, and toileting. This comprehensive approach promotes independence, engagement, and social relationships and aligns with the shift from a medical model to a social model of disability. Service providers use a variety of strategies to promote a child’s participation, including authentic assessments [32], interest-based intervention [33], assistive technology [31], and collaborative teaming [34], all within a family-centered framework [35,36].

In order to implement contemporary strategies that ensure a child has the opportunity to access these life situations, service providers require knowledge and skills that enable them to effectively collaborate with family and professional team members to create team interventions that promote inclusion, participation, and belonging. Because they play such a critical role in fostering participation and promoting belonging, service providers need appropriate *support* and resources, such as training and collaboration with other professionals, to implement comprehensive and interactive services.

Most service providers have been trained under the medical model of disability in which impairments are identified, and remediation is provided. The movement toward inclusion, participation, and belonging requires a shift to the social model of disability, which identifies the interaction between a person’s characteristics, their physical and social environment, and the context [37]. This social model, consistent with the variety of international commitments previously discussed, emphasizes the need to promote inclusion, participation, and belonging. Achieving this goal requires service providers to enhance their knowledge, skills, and sometimes attitudes to work effectively within a flexible, team-based service provision model.

Providers should align their strategies and goals to those detailed in UNICEF’s recently published DIPAS, which include preventing stigma and discrimination, creating disability-inclusive and accessible services and programs, and facilitating full participation in all contexts. By following the lead of international organizations like UNICEF, providers can contribute to creating a more equitable society for children with disabilities.

## 5. Conclusions

Inclusion, participation, and belonging are crucial to the development of children with disabilities. This paper has explored the importance of creating environments that are inclusive, value diversity, promote active participation, and foster belonging for all children. Contemporary research indicates the benefits of inclusive practices, effective strategies to enhance participation, and the significance of cultivating a sense of belonging.

Inclusive practices can have a transformative impact on the lives of children with disabilities; however, inclusion is only the first step on the path to belonging. Inclusive environments provide opportunities for active participation in activities that occur within those environments and ensure that children create social relationships based on shared interests, contributing to growth, development, and independence.

The authors highlight the significance of active participation as a catalyst for learning, growth, and empowerment. By creating environments that encourage children’s engagement and involvement, service providers can enable children to build on their strengths and facilitate their development across multiple domains. Tailoring activities to match their interests, providing necessary services, and promoting autonomy are key strategies to enhance participation and overall well-being.

We emphasize the importance of promoting belonging in children with disabilities. Feeling accepted, valued, and connected to their communities is essential for a person’s emotional well-being and social integration. Creating inclusive spaces, nurturing positive relationships, and recognizing the unique contributions of each child enables service providers to cultivate an environment where children with disabilities belong and thrive. We recognize, however, that more research is needed to understand the complexity of belonging and to create programming that promotes belonging and equal opportunities for all. Components of belonging, as described by Mahar, Allen, and Renwick, may be useful when elucidating how and under what circumstances they interact, helping us create systems that support and facilitate belonging.

The inclusion, participation, and belonging of children with disabilities are fundamental rights that must be upheld. Service providers, parents, and caregivers can contribute to the holistic development of children with disabilities by implementing effective strategies and embracing the principles of inclusion. The collective efforts of service providers and families are crucial for creating an equitable society where all children, regardless of their abilities, can participate, belong, and reach their full potential.

## Figures and Tables

**Table 1:** United States commitments to inclusion.

Law	Purpose
Section 504 of the 1973 Rehabilitation Act [10]	Prohibits discrimination against individuals with disabilities by organizations and employers that receive federal funding. Applies to a wide range of entities, including schools, colleges, universities, healthcare providers, government agencies, and private employers.
Individuals with Disabilities Education Act of 1975 [11]	Ensures that children with disabilities have access to a free and appropriate public education (FAPE) in the least restrictive environment (LRE). Students with disabilities should be educated with their non-disabled peers in the general education classroom as much as possible; they should not be separated unless learning cannot be achieved, even with aids and services. Also includes provisions for early intervention services for infants and toddlers with disabilities.
American with Disabilities Act of 1990 [12]	Protects the rights of individuals with disabilities in many areas of public life, including employment, public accommodations, transportation, telecommunications, and government services. Requires equal access, reasonable accommodations, and non-discrimination for those with disabilities.

**Table 2:** United Nations commitments.

Commitment	Purpose
Convention on the Rights of the Child (1989) [15]	Defines childhood as a period separate from adulthood and vows to protect children’s rights as they learn, grow, and play.
Convention on the Rights of Persons with Disabilities (2006) [2]	The International human rights treaty is committed to safeguarding the rights and dignity of all people with disabilities. Includes several articles dedicated explicitly to the rights of women and children with disabilities.
Sustainable Development Goals (SDGs) (2015) [16]	Seventeen global goals aimed at addressing various social, economic, and environmental challenges. Disability is included in all aspects of SDGs, specifically goals of education, employment, economic growth, accessibility, and equality.
UNICEF’s Disability Inclusion Policy and Strategy (DIPAS) 2022–2030 [17]	Prioritizes the inclusion of children with disabilities in UNICEF’s programs, policies, and initiatives. Ensures that children with disabilities are not left behind in access to education, health, protection, water, and sanitation.
UNICEF’s Core Commitments for Children in Humanitarian Action (CCC) (2020) [18]	Ensures that the rights and well-being of children, including those with disabilities, are prioritized and protected during emergencies and crises.
UNESCO Salamanca Statement (1994) [19]	Produced during the UNESCO World Conference on Special Needs Education, emphasizing the principle of inclusive education. Calls for inclusive schools to be the most effective means of combating discriminatory attitudes, building an inclusive society, and achieving education for all (EFA).
UNESCO Dakar Framework (2000) [20]	Reiterated the commitment of the EFA initiative, emphasizing inclusive education as fundamental to achieving quality education for all children, particularly those marginalized, vulnerable, or with disabilities.
UNESCO Incheon Declaration (2015) [21]	Recognizes inclusive education as a transformative approach to promote learning and participation for all.
